# Ultrasound-guided serratus anterior plane block for transapical transcatheter aortic valve implantation

**DOI:** 10.1186/s13019-023-02125-4

**Published:** 2023-01-06

**Authors:** Ling Peng, Meng Ding, Wei Wei

**Affiliations:** Department of Anesthesiology, West China Hospital, Sichuan University, 37 Guo Xue Xiang, Chengdu, 610041 China

**Keywords:** Serratus anterior plane block, Transapical transcatheter aortic valve implantation, Analgesia

## Abstract

**Background:**

Reducing postoperative pain due to the thoracotomy incisions in patients undergoing transapical transcatheter aortic valve implantation remains a challenge.

**Methods:**

We introduced ultrasound-guided serratus anterior plane block (SAPB) in a patient with severe aortic insufficiency and chronic obstructive pulmonary disease before surgical intervention.

**Results:**

The patient’s postoperative 1 h, 4 h, and 12 h resting visual analogue scale scores were 3, 1, and 1 without single injection of morphine or dezocine for rescue analgesia.

**Conclusions:**

Ultrasound-guided SAPB could improve analgesia after transapical transcatheter aortic valve implantation.

To the editor

Transcatheter aortic valve implantation (TAVI) has become an alternative treatment for aortic valve disease in elderly or high-risk patients [1]. However, reducing postoperative pain due to the thoracotomy incisions remains a challenge in transapical access TAVI [2, 3]. Although regional anesthesia including epidural analgesia and paravertebral nerve block are effective, they can be difficult to perform and increase the risk of pneumothorax and epidural hematoma because TAVI procedure requires heparinization [4].

Ultrasound-guided serratus anterior plane block (SAPB) may be a promising regional analgesia method for patients undergoing transapical TAVI. Blanco R et al. demonstrated that SAPB can block the lateral cutaneous branches of the thoracic intercostal nerves from T2 to T9 for 750–840 min [5]. The SAPB has been reported as effective for analgesia in mastectomy and video assisted thoracic surgery [5].

Written informed consent was obtained both for the block intervention and publication of the reports. A 79-year-old male patient (weight: 70 kg, height: 172 cm) with chronic obstructive pulmonary disease was scheduled for TAVI due to severe aortic insufficiency. Transapical access was chosen because therosclerotic plaque in the femoral artery was indicated by preoperative vascular computed tomographic angiography. Before general anesthesia induction, the SAPB was performed on the left under ultrasound guidance (Anaesus ME7, Mindray Bio-Medical Electronics, Shenzhen, China). A high-frequency linear probe was placed in the mid-axillary line to identify the fifth and sixth ribs, the latissimus dorsi muscle, and serratus anterior muscle in a sagittal plane. A 22-gauge, 50 mm needle (Stimuplex^Ⓡ^ D B.Braun, Melsungen, Germany) was inserted in-plane (Fig. 1A). A total of 100 mg (30 ml 0.33%) ropivacaine was injected above the serratus anterior muscle and below the serratus anterior muscle (Fig. 1B). The local anesthetics linear spread between the latissimus dorsi muscle and serratus anterior muscle and between the serratus anterior and ribs, respectively. An apical access was established through a left mini-thoracotomy (4–5 cm) (Fig. 1C). After surgery, flubifprofen axetil injection (50 mg) and tropisetron hydrochloride injection (5 mg) was routinely administrated for analgesia and antiemetic, respectively. A standardized postoperative patient controlled intravenous analgesia (PCIA) pump was connected. The PCIA pump was set up with sufentanil 300 μg, dexmedetomidine 200 μg, and granisetron 9 mg in sodium chloride 0.9%, 200 ml at 2 ml/h, with a single bolus dose of 0.5 ml, and lockout time of 15 min.

**Fig. 1:**
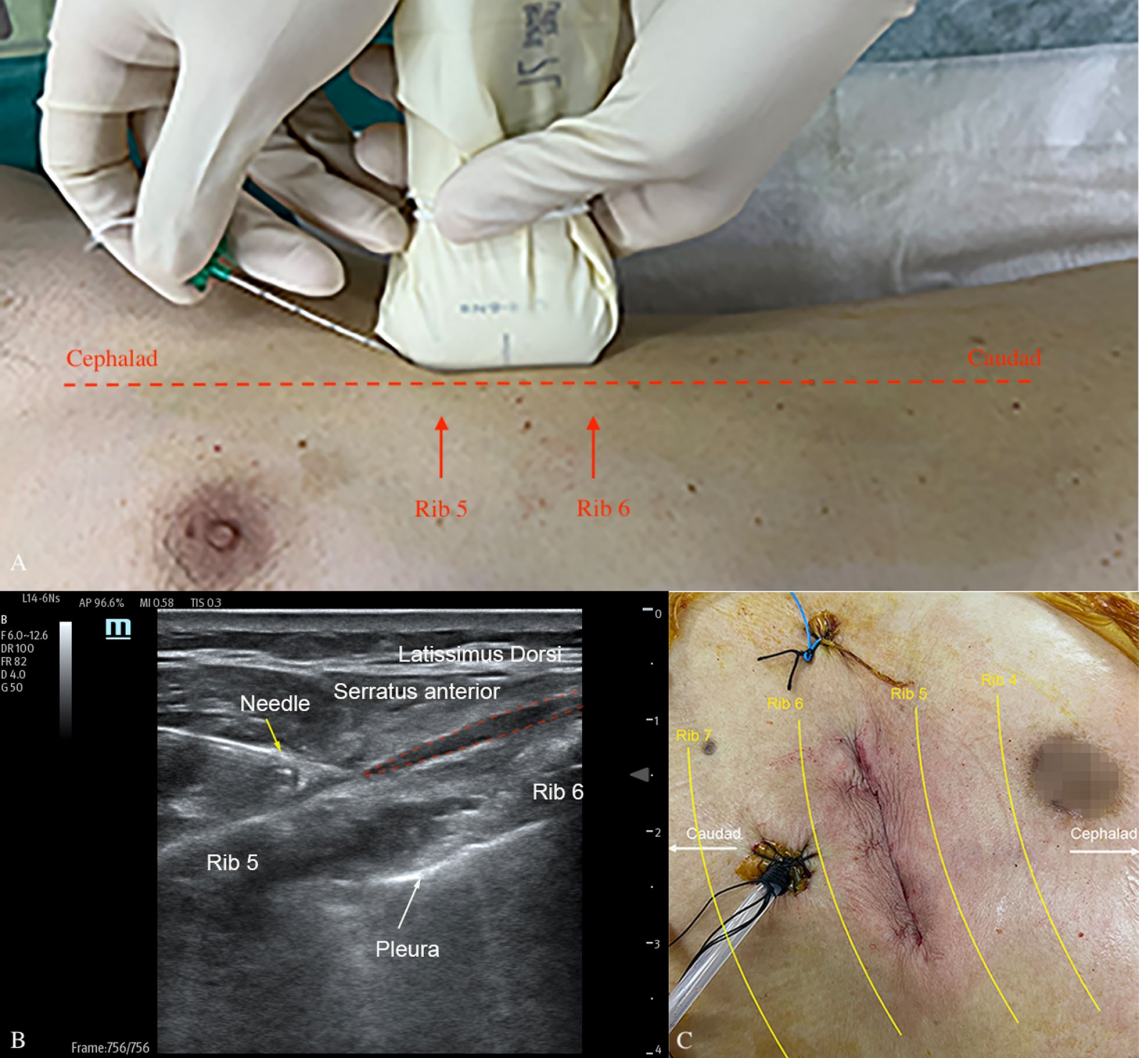
Serratus anterior plane block. **A** Location of administration of the serratus anterior plane block. **B** Ultrasound image of the serratus anterior plane block. The yellow arrow indicates the needle. The area of red dotted line indicates the spread of local anesthetics. **C** The left mini-thoracotomy into left ventricular apex

The patient was extubated in the operating room 8 min after surgery and transferred to the intensive care unit. Postoperative 1 h, 4 h, and 12 h resting visual analogue scale (VAS) scores were 3, 1, and 1 without single injection of morphine or dezocine for rescue analgesia. No complications associated with the SAPB were observed. The patient was transferred to ward on postoperative day 1 and discharged on postoperative day 4.

The pain of left mini-thoracotomy could increase postoperative complications and delay recovery in patients underwent transapical TAVI [4, 6]. Optimal management of postoperative pain hence is necessary to prevent complications and to enhance recovery. Compared with other regional anesthesia, such as paravertebral blockade, thoracic epidural anesthesia, and pectoralis nerve plane block, the SAPB is priority with easy operation and low risk of epidural hematoma. Therefore, we used SAPB for a specific type of minimally invasive cardiac surgery, transapical TAVI. The efficacious of SAPB in post-thoracotomy pain management has been demonstrated both in pediatric and adult cardiac surgery [7–9]. Edwarts JT et al. suggested that both superficial and the deep SAPB can be used for postoperative analgesia [10]. Continuous deep SAPB was also effective for analgesia in mitral valve surgery via right minithoracotomy [11]. In our case, the VAS scores were lower than 3 within 12 h after surgery indicated that combined superficial and deep SAPB was effective. However, the efficiency of different SAPB infusion techniques in patients undergoing transapical TAVI needs further studies.

Ultrasound-guided SAPB is feasible in conjunction with a postoperative multimodal analgesia in patients undergoing transapical TAVI. Perspective randomized controlled studies are needed to further demonstrate its efficiency and safety.

## Data Availability

All data generated or analyzed during this study are included in this published article.

## Abbreviations

TAVI: Transapical transcatheter aortic valve implantation
SAPB: Serratus anterior plane block
PCIA: Patient controlled intravenous analgesia
VAS: Visual analogue scale
